# Stress as worsening of the signs and symptoms of the geographic tongue during the COVID-19 pandemic: a pilot study

**DOI:** 10.1186/s12903-022-02609-0

**Published:** 2022-12-03

**Authors:** Miriam Beatriz Jordão Moreira Sarruf, Valquiria Quinelato, Guilherme Jordão Moreira Sarruf, Henrique Eduardo Oliveira, Jose Albuquerque Calasans-Maia, Hilka Quinelato, Telma Aguiar, Priscila Ladeira Casado, Larissa Maria Assad Cavalcante

**Affiliations:** 1 Dentistry School, Universidade Federal Fluminense, Mario Santos Braga St, 28, Center, Niterói, RJ 24020-140 Brazil; 2 Inter-American Faculty of Social Sciences (FICS), Asunción, Paraguay

**Keywords:** Coronavirus, Coronavirus infection, Stress, Geographic tongue

## Abstract

**Background:**

Geographic Tongue (GT) is a benign inflammatory disorder of unknown etiology, which is characterized by the loss of epithelium due to the atrophy of filiform papillae. It usually occurs on the dorsum of the tongue and may extend to its lateral edges. It appears as an erythematous area surrounded by whitish and slightly elevated margins. In most cases, the condition is asymptomatic**,** although some individuals may report symptoms that include a burning sensation of the tongue.

**Objective:**

Assess whether there was a change in the clinical aspect of Geographic Tongue (GT) during the COVID-19 pandemic.

**Methods:**

Thirty-two participants were recruited from Dentistry School Universidade Federal Fluminense. Anamnesis and oral examination were performed to collect medical history. The participants were split into two groups: control group (no GT) n = 20 and test group (with GT) n = 12. In the second step, nine participants from a 12 (75%) of the test group were contacted by phone and answered a questionnaire about changes in the signs and symptoms of GT during the pandemic. The subjects were subdivided into two groups: GT with and without signs and symptoms exacerbation.

**Results:**

In the first phase of the research, no statistical difference between control and test groups was observed regarding clinical criteria such as age (p + 0.72), gender (*p* = 0.24), and systemic diseases (*p* = 0.58). In the second phase, there was a statistical difference between GT groups with or without symptom exacerbation in terms of age and stress as a factor of the oral symptoms (*p* = 0.3 and 0.2), respectively. Younger patients showed a worsening of the oral lesions related to GT (*p* = 0.3) and reported stress during the pandemic (*p* = 0.02).

**Conclusion:**

Younger patients were more susceptible to stress and presented more exacerbation of the oral lesions related to GT.

## Background

Geographic Tongue (GT), also described in the literature as glossitis migrans, erythema migrans, benign migratory glossitis, or stomatitis areata, is an inflammatory disorder, generally asymptomatic of unknown etiology, which commonly occurs on the dorsum of the tongue and can sometimes involve its lateral borders. Its clinical presentation is often described as an erythematous area, which occurs due to filiform papillae´s atrophy and consequent epithelium thinning [1, 2], surrounded by whitish and slightly elevated margins, resembling a map-like appearance. However, in this condition, the fungiform papillae are not affected by this kind of atrophy [3].

The exact cause of atrophy of the tongue’s papillae in patients with GT remains unknown. However, it has been shown that the tongue epithelium is continually renewed by proliferation, maturation, and apoptosis processes, which are partly controlled by the epidermal growth factor (EGF) [4]. Occasionally, lesions are found in other oral cavity places, called migratory stomatitis, geographic stomatitis, or ectopic GT [5, 6]. Studies have shown a significant association between GT and fissured tongue (FT) [7].

GT can be clinically classified as active or typical when a whitish, slightly high margin demarcates it; passive, abortive, or atypical when this border disappears before its formation process is finished [2]. It is a dynamic lesion that presents morphology changes, can heal in spontaneous ways periodically and has a recurrent character in different moments [8].

Recently, GT was classified by Picciani et al. [9] according to its severity. The criteria established by the authors described the severity of the lesion according to the following aspects: As mild, they considered those which showed slightly erythematous areas surrounded by a thin halo which can be present or not, affecting only small areas of the tongue; as moderate (mild to severe) they classified the lesions which presented erythema, surrounded by thick whitish halo, being or not associated with Fissured Tongue (FT) and of variable extension; and as severe they included the lesions with intense erythema, wide whitish halo, associated with FT, affecting a large extension of the tongue, mainly the dorsum [9].

The prevalence varies from 0.2 to 14% in some studies [10], however other research reports this prevalence in a range of 1% to 2.5% [5, 11]. This variation in the reported rates may occur due to the difference between the population examined, types of examination performed, and diagnostic criteria [12]. GT is more often seen in adults than in children [13]. Some studies prefer the female gender [14], while others do not differentiate between genders [15].

GT has been described in patients with a family history of the same condition and is often found in individuals with immune disorders such as psoriasis, in the presence of stress and/or anxiety, and in allergic or atopic conditions [16]. Recently, celiac disease and iron deficiency anemia have been observed in patients with GT [17].

As main symptoms, pain, burning sensation, and discomfort are occasionally reported by some individuals [3, 6, 18] and can be treated with mouthwashes containing anesthetic, and/or antihistamines, and/or topical corticosteroids [3].

Although no specific treatment protocol exists, some drugs, such as retinoic acid, cyclosporine, antihistamines, tacrolimus, and immune system regulators, have been used to treat GT to modulate the inflammatory response [19].

Anxiety is significantly present in GT patients among the psychological factors associated with the condition [20]. Studies report stress and depression as factors related to the lesions’ appearance [21].

On March 11, 2020, COVID-19 was officially declared by the World Health Organization (WHO) as a Pandemic [22]. Measures to prevent the virus spread included confinement of more than half of the world’s population, closure of schools and universities, and social distancing. A state of emergency was also adopted in several countries [23–25].

Humanity faced uncertainty and has experienced sudden changes in daily life due to the novel coronavirus Pandemic (COVID-19). Along with its high infectivity and mortality rates, Corona Virus Disease had a tremendous psychological impact all around the world resulting in mass hysteria, economic burden, and financial losses. The quarantine imposed on the citizens to fight COVID-19 represented by blocks in all countries, has generated panic, anxiety, and depression. In the future we should expect reports of, post-traumatic stress disorder (PTSD) as another symptom developed due to the Corona Virus Disease [26, 27].

In addition to the most common symptoms of severe acute respiratory syndrome coronavirus 2 (SARS-CoV-2) infection such as myalgia, dry cough, fatigue, gastrointestinal changes, sore throat, headache, hyposmia, sinonasal inflammation, anosmia, hypogeusia, dyspnea, and pneumonia, [28–32], mucocutaneous manifestations such as geographic tongue, traumatic ulcer, generalized mucositis, angular cheilitis, fungal infection (candidiasis), erosions, papillary hyperplasia, herpes simplex, angina bullosa, have also been reported in patients with COVID-19 [33–36].

Subramaniam et al. [35] performed an observational study, and 713 patients positive for COVID-19 were evaluated. Nine presented mucocutaneous manifestations; three were diagnosed with geographic tongue. The literature has shown several cases of patients positive for COVID-119 and the presence of GT associated with other oral lesions [33, 35, 37].

Although its etiology remains unknown, studies show that genetic factors, allergies, vitamin deficiency, psoriasis, stress, and depression may be associated with GT [16, 21]. Furthermore, some studies demonstrate oral lesions in patients during the COVID-19 pandemic [33, 37, 38]. Based on this, this study’s scientific question is: was there an exacerbation of GT signs and symptoms caused by the pandemic stress or by COVID-19? Therefore, this study’s objective was to assess whether there was a change in the clinical aspect of GT or a worsening of its symptoms during the COVID-19 pandemic.

## Methods

This study is a descriptive, observational, cross-sectional case–control study approved on November 2, 2020, by the Research Ethics Committee of Hospital Universitário Antonio Pedro (HUAP) under the number 4.376.011. The informed consent form was obtained and signed by all research participants of the study. The Strengthening the Reporting of Observational Studies in Epidemiology (STROBE) [39] was followed for the development of its design. The methodology of the present study was divided into two stages:

*1st stage* clinical diagnosis of the geographic tongue through the routine physical examination of the oral cavity.

Research participants over 18 years old were randomly recruited from the Oral Diagnosis Discipline of UFF Dentistry School for six months. They were submitted to a detailed anamnesis. Their identification data, medical history, and information concerning systemic and autoimmune diseases, smoking habits, alcohol, and medication use were registered in the clinical record. Subsequently, the participants underwent a stomatological examination.

**Fig. 1:**
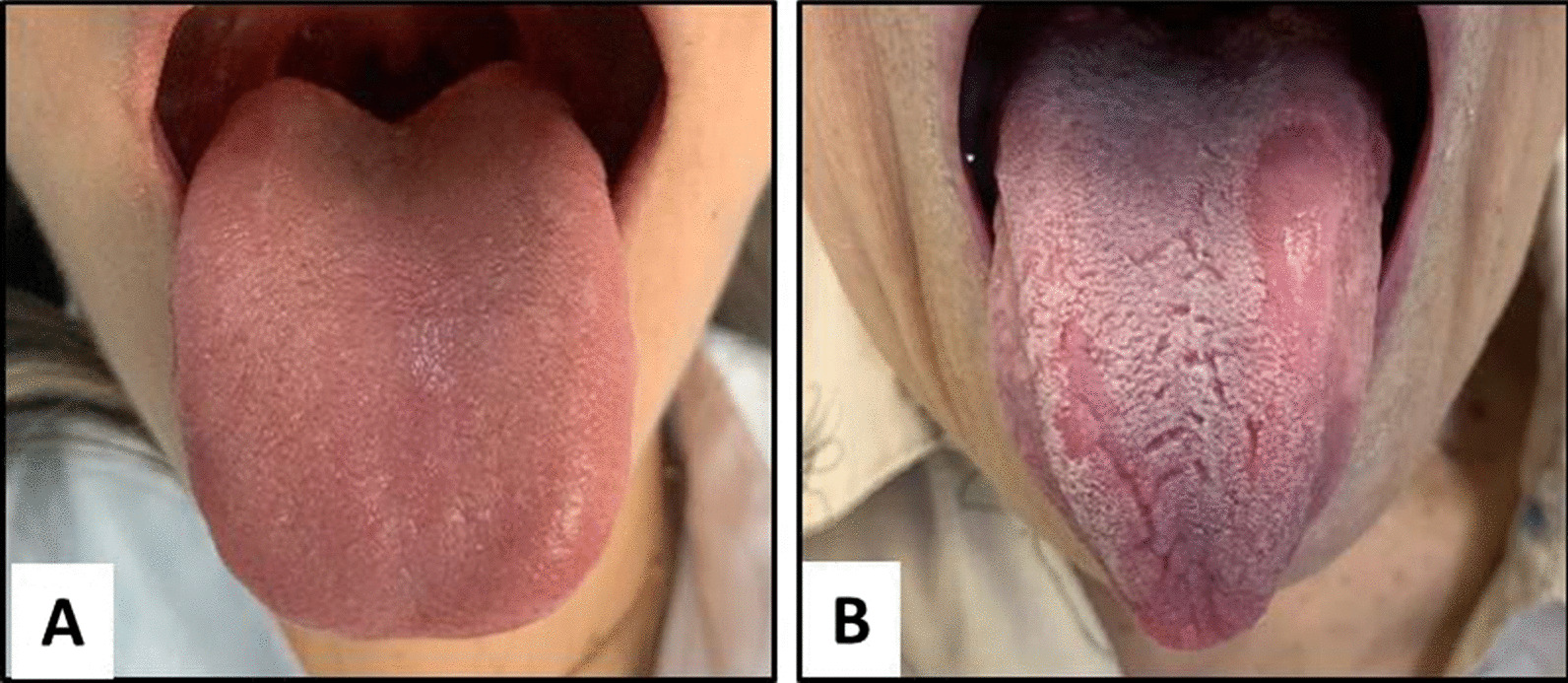
Photographic images exemplify each research group. **A**. Photograph of Control Group tongue. **B**. Photograph featuring a geographic tongue of Test Group

After the anamnesis and intraoral examination of the participants, two groups were formed: (A) Control Group (absence of GT), n = 20; (B) Test Group (with GT), n = 12 (Fig. 1A and B).

### Inclusion criteria

- Patients of both genders over 18 years old
- Patients with Geographic Tongue (Test Group) and Without Geographic Tongue (Control Group)
- Patients without a history of macro trauma in the region where the material should be collected.
- Patients who have agreed to sign the informed consent term.

### Exclusion criteria

- Patients who have psoriasis
- Carriers of autoimmune diseases.

*2nd stage* The Test Group (with GT) participants were contacted by telephone and answered a questionnaire about the occurrence of changes in the signs and symptoms of GT during the novel coronavirus pandemic, which consisted of the following questions: have you presented symptoms of Sars-Cov-2 infection? Have you been tested for coronavirus? Which test was performed? Was the test result positive? Did you take any medication for COVID-19 treatment? Have you noticed changes in the aspect, any new symptoms, or worsening of the symptoms of the GT? Have you associated the changes (signs and symptoms) with any factor?

The GT Severity was classified according to the GT Severity Index: Mild, Moderate or Severe according to Picciani et al. [9].

### Statistical analysis

The numerical variables were expressed as mean ± standard deviation, and the Normality Test (Shapiro–Wilk Test) was applied: *t*-test (normal sample) and Mann–Whitney Test (non-normal sample) were used. Nominal variables were assessed using the chi-square test, including setting the odds ratio with a 95% confidence interval. The *p*-value < 0.05 was considered statistically significant. The Microsoft Office 2013 Excel program was used for data tabulation and the Prisma GraphPad 6.0 software (GraphPad Software, La Jolla, CA-EUA) for statistical calculations.

## Results

A total of 140 patients were evaluated. Among these, 32 participants were included in this study, 12 from the Test Group (geographic tongue) and 20 from the Control Group. In Test Group, 4 participants reported burning symptoms associated with the ingestion of spicy or citrus drinks/foods. Stress was declared as the lesion’s onset by 3 participants (Table 1).

**Table 1:** Clinical characteristics of the Test Group (geographic tongue)

Parameters	N = 12	(%)
*Site of the lesion*		
Dorsum	3	25.00
Lateral borders	2	16.67
Dorsum and lateral borders	4	33.33
Apex of the tongue	1	8.33
Passive lesions	2	16.67
*Number of lesions*		
1 lesion	3	25.00
2 lesion	3	25.00
3 lesion	2	16.67
4 lesion	2	16.67
Passive	2	16.67
*Severity index*		
Mild	5	41.67
Moderate	4	33.33
Severe	1	8.33
*Factor related to the lesion onset*		
Stress	3	25.00
Absent	9	75.00
*Symptoms*		
Burning	5	41.67
Absent	7	58.33
*Triggering factors for the symptoms*		
Drinks	1	8.33
Drinks/citrus fruits	1	8.33
Spicy foods/citrus fruits	2	16.67
Spontaneous	1	8.33
No symptoms	7	58.34

There were no statistical differences between the groups regarding gender (*p* = 0.74) when the clinical data of the Test Group (GT) were compared with Control Group using the chi-square test (Table 2). Fifteen (46,87%) patients included in the study declared to use medicines (antihypertensive, antihyperglycemic, antacid, antiviral, lipid-lowering, contraceptive, vitamin D, calcium and antidepressant), and 14 (43,75%) reported to have systemic diseases (hypertension, hyperglycemia, osteoporosis, gastritis, hepatitis C and depression) (Table 2).

**Table 2:** Clinical data

Parameters	Control N = 20	Geographic tongue N = 12	*p-value (OR;CI)*
*Age*			
Mean	45 ± 15.90	46.91 ± 16.95	0.72
*Gender*			
Male	5 (25.00%)	1 (8.33%)	0.24
Female	15 (75.00%)	11 (91.67%)	(3.67;0.37–36.00)
Smoker	2 (10.00%)	1 (8.33%)	0.84 (1.29;0.10–16.05)
Alcohol consumption	6 (30.00%)	2 (16.67%)	0.40 (2.14;0.36–12.90)
Use of drugs	1 (5.00%)	0 (0.00%)	0.43 (1.92;0.07–51.07)
Medical treatment	7 (35.00%)	4 (33.33%)	0.92 (1.08;0.24–4.88)
Medication use	9 (45.00%)	6 (50.00%)	0.78 (0.82; 0.19–3.34)
Allergy	1 (5.00%)	2 (16.67%)	0.27 (0.26;0.02–3.27)
Systemic diseases	8 (40.00%)	6 (50.00%)	0.58 (0.67;0.16–2.82)
Fissured tongue	0 (0.00%)	2 (16.67%)	0.05 (0.10;0.00–2.34)

Chi-square test + *t* test

Among the 12 participants with GT included in the study, 9 were contacted in the second stage of the research. Contact with the others was not possible due to incorrect telephone data. In the comparison between groups with and without exacerbation of oral manifestations during the pandemic, there was no difference in gender (*p* = 0.34) or the positive test for COVID-19 (*p* = 0.63). However, a statistical difference was observed in the age (*p* = 0.03) associated with stress as an exacerbation factor for symptoms of the GT (*p* = 0.02) (Table 3). Among the three participants with GT who took the swab test with positive result for COVID-19, only one presented a change in the lesion`s clinical aspects and reported that the pain on her tongue became spontaneous, especially on the dorsum, which also presented bleeding.

**Table 3:** Analysis of the oral manifestations of the Test Group during the COVID-19 pandemic

Parameters	Geographic tongue without exacerbation of the oral symptoms during the COVID-19 pandemic (N = 5)	Geographic tongue with exacerbation of the oral symptoms during the COVID-19 pandemic (N = 4)	*p*-valor (OR;CI)
*Gender*			
Male	1 (20.00%)	0 (00.00%)	0.34
Female	4 (80.00%)	4 (100.00%)	(3.0;0.094–95.25)
*Age*			
Mean	49,00 ± 9,70	29.50 ± 6.87	0.03
*Symptoms for COVID19*			
Yes	2 (40.00%)	1 (25.00%)	0.63
No	3 (60.00%)	3 (75.00%)	(2.00;0.11–35.83)
*Tested for COVID-19*			
Yes	2 (40.00%)	1 (25.00%)	0.63
No	3 (60.00%)	3 (75.00%)	(2.0;0.11–35.83)
*Positive test for COVID-19*			
Yes	2 (40.00%)	1 (25.00%)	0.63
No	3 (60.00%)	3 (75.00%)	(2.0;0.11–35.83)
*Medication use for COVID-19*			
Yes	2 (40.00%)	1 (25.00%)	0.63
No	3 (60.00%)	3 (75.00%)	(2.0;0.11–35.83)
*Report of stress during the pandemic*			
Yes	1 (40.00%)	4 (100.00%)	0.02
No	4 (60.00%)	0 (00.00%)	(0.3; 0,00–1.18)
*Use of medication for GT symptoms*			
Yes	0 (00.00%)	1 (25.00%)	0.23
No	5 (100.00%)	3 (75.00%)	(0.21;0.00–6.82)

Chi-square test + Mann Whitney test

## Discussion

Geographic Tongue (GT) is an inflammatory disorder, usually asymptomatic, of unknown etiology, which regularly happens at the tongue’s dorsum and may sometimes involve its lateral borders. Erythematous areas characterized the clinical aspect of GT with a whitish, slightly high margin due to atrophy of the filiform papillae [1, 2]. The lesion is often found in people with immunological sickness inclusive of psoriasis, withinside the presence of stress and/or anxiety, and may be related to allergic or atopic conditions [16].

On November 14, 2020, more than 53.78 million confirmed cases of COVID-19 were recorded worldwide, and more than 1.3 million deaths related to the disease have occurred [22]. This situation has caused a global impact of significant proportions. Today, the world is affected by the stress, anguish, and panic resulting from the COVID-19 pandemic [26, 27]. In the present study, 4 participants associated the worsening of their signs and symptoms of GT with the stress caused by the COVID-19 pandemic. These signs and symptoms were generalized and were reported by patients as more intense and more frequent burning, spontaneous burning, itchy, and tongue bleeding. However, the positive patient for COVID-19 was more affected.

Late in the 19th and mid-twentieth centuries, several studies suggested emotional stress as a possible etiological factor associated with GT [40, 41]. Later, Redman et al. [42] investigated this possibility and showed the prevalence of the lesion in psychiatric patients compared to university students and concluded that stress might be one of the factors that influence the onset of GT and its exacerbation in susceptible individuals.

Recent studies have demonstrated the association of GT to the presence of anxiety, stress, and depression [20, 38], corroborating the results of this study, where stress was statistically related to the worsening of the symptoms of geographic tongue. Furthermore, it was observed that younger patients were more susceptible to stress and, consequently, developed exacerbation or worsening of their symptoms. This is in line with the results of Wieckiewicz et al. [43] which demonstrated that female patients under the age of 28.five years had increased levels of parafunctional oral habits, anxiety, depression, and headache during the COVID-19 pandemic.

In addition to geographic tongue, other forms of oral lesions have been observed in patients diagnosed with COVID-19 ranging from small ulcers to large vesicles, glossitis, candidiasis, herpes simplex, and generalized mucositis [35, 36, 44]. Another symptom of coronavirus disease 2019 is headache, one of the maximum prevalent (about 70%), that is disabling and represents a continual symptom hard to be treated. It was related to innate immune reaction to SARS-CoV-2, therefore future research is important to clarify this pathway [32, 45, 46].

Subramaniam et al. [35], in an observational study, described that these mucocutaneous manifestations are not secondary manifestations of COVID-19, but coincident presentations due to local causes or other reasons such as systemic conditions (nutritional deficiency, anemia, diabetes) or stress caused by the pandemic itself. Therefore the systematic review that analyzed mucocutaneous manifestations in a COVID-19 and highlighted that more than 50% of the COVID-19 case preceded or appear simultaneously with the symptoms of infection by SARS-CoV-2, suggesting an association, and not being a random factor, between viral infection and oral manifestations [36].

Other important factors in SARS-CoV-2 infection are the exacerbation of autoimmune sicknesses, such as herpes simplex virus, and varicella-zoster virus infections with inside the oral cavity. It continues to be doubtful whether or not signs and symptoms are manifestations of the disease or arise because of the lack of immune response [47]. The oral manifestations associated with COVID-19 are real, therefore the intraoral examination is important to be performed in all patients with COVID-19 [38, 48].

Besides GT, other oral lesions were related to stress [45, 46]. Gallo et al. [45] conducted a study with 50 patients with Recurrent Aphthous Stomatitis (RAS), one of the most frequent ulcerated lesions in the oral cavity. They concluded that psychological stress played an essential trigger for the appearance of these lesions in their patients. Stress induces an immunoregulatory activity by increasing the number of leucocytes at the site of inflammation. This characteristic is observed in the pathogenesis of RAS [49]. Another lesion in which exacerbation was related to stress is Lichen Planus [46, 50]. Burning Mouth Syndrome (BMS) has also been strongly associated with psychogenic factors such as stress, depression, and anxiety [51]. As the main symptoms of GT, burning, pain, and discomfort are also occasionally reported [3]. In our study, 5 participants reported burning on their GT.

Literature data has demonstrated a higher incidence of GT on the tongue’s dorsum [1]. In the present study, three lesions were observed on the dorsum and four on the dorsum extending to the lateral borders. The remaining cases include lesions on the lateral border and one lesion on the apex of the tongue.

Its clinical characteristics have been described as erythematous areas with whitish and slightly elevated margins [1, 7] and can be classified as active or passive [2]. This study showed that 8 patients in the sample had the clinical characteristics described above. Two cases presented as passive GT. Two associations with Fissured Tongue were also observed. The literature has reported this association, describing fissured tongue as areas of stagnation on the tongue’s surface where glossitis can start [52].

According to the recently published GT Severity Index [9], our study showed five cases classified as mild, four as moderate, and one as severe GT.

GT has been described in patients with a family history of the same condition and frequently occurs in individuals with immunological and some systemic diseases [16]. In the present study, two patients, a mother and a daughter, presented the condition. Although a pattern of heredity has been suggested as a form of polygenic transmission, changes in the lingual microbiota profiles have been observed. However, it is unknown whether this finding is a consequence of the lesions or is associated with the disease’s initial or progression factors [53]. Studies reported a higher prevalence of GT in females [14], corroborating this research’s data.

The prevalence of GT in the female gender was confirmed in the sample studied. Burning was not a significant symptom in the first stage of the research, but the worsening of this symptom and changes in the clinical aspects of GT were observed during the COVID-19 pandemic. At this second stage of the study, most participants have associated stress with either exacerbating symptoms or changing the clinical presentation of lesions. Stress was more prevalent in younger participants. Therefore, some limitations of this research are highlighted, such as the absence of stress quantification in the population studies, the small number of patients included in the study, and the inclusion of one patient that was using antidepressive in the test group. The studies on oral lesions related to COVID-19 remain at an early stage. Therefore, despite the worsening of the apparent symptoms of GT associated with COVID-19 pandemic stress, it is crucial to perform an accurate oral examination and stress evaluation in patients affected by Sars-Cov-2 to understand oral manifestations of this new disease in the world.

In addition to oral manifestations of COVID-19 infection, the virus itself can be detected in saliva. Angiotensin-converting enzyme 2 (ACE2), which allows the attachment of the virus, is also present in the oral cavity [47, 54, 55]. Would this justify the more severe oral symptoms of GT in the COVID-19-positive patient reported in this study?

Diagnostic methods focused on the detection of SARS-CoV-2 in saliva have been investigated, but the studies are still in the validation stage of the methods [54, 55].

## Conclusion

Younger patients were more susceptible to stress and, consequently, presented exacerbation of the oral lesions related to GT. This pilot study shed a red light on the stress caused by the COVID-19 pandemic in younger adults; in addition to the development/exacerbation of oral lesions, other systemic diseases also can emerge/aggravate, due to increased stress, such as acute myocardial infarction and stroke.

## Data Availability

The datasets generated and/or analyzed during the current study are available from the corresponding author on reasonable request.

## Abbreviations

COVID-19: Coronavirus disease 2019
GT: Geographic Tongue
SARS-CoV-2: Severe acute respiratory syndrome coronavirus 2
WHO: World Health Organization
